# Simulated Workflow Feasibility Evaluation of a Web-Based Periorbital Measurement Platform: Development and Usability Study

**DOI:** 10.2196/82859

**Published:** 2026-04-17

**Authors:** George R Nahass, Jacob van der Ende, Sasha Hubschman, Benjamin Beltran, Bhavana Kolli, Caitlin Berek, James D Edmonds, RV Paul Chan, Pete Setabutr, James W Larrick, Darvin Yi, Ann Q Tran

**Affiliations:** 1 Department of Biomedical Engineering University of Illinois Chicago Chicago, IL United States; 2 Department of Ophthalmology University of Illinois Chicago Chicago, IL United States; 3 Quina Care San Miguel Ecuador; 4 Panorama Research Institutes Sunnyvale, CA United States

**Keywords:** artificial intelligence, global health, deployable applications, periorbital distance, simulation

## Abstract

**Background:**

Periorbital measurements such as margin to reflex distances, palpebral fissure height, and scleral show are critical in diagnosing and managing conditions like ptosis and disorders of the eyelid. However, deployment of automated periorbital measurement algorithms in structured research workflows remains limited by the lack of integrated capture and data management infrastructure.

**Objective:**

We developed and evaluated Glorbit, a lightweight, browser-based application for automated periorbital distance measurement using artificial intelligence (AI). The objective was to evaluate end-to-end workflow feasibility of the platform under simulated, operator-run conditions.

**Methods:**

The application integrates a DeepLabV3 segmentation model into a modular image processing pipeline with secure, site-specific Google Cloud storage, supporting local preprocessing and cloud upload through Firebase-authenticated logins. The full workflow—metadata entry, facial image capture, segmentation, and upload—was tested. After the session, the participants completed a Likert-style survey.

**Results:**

Glorbit successfully ran on all tested platforms, including laptops, tablets, and mobile phones across major browsers. A total of 15 volunteers were enrolled in this study in which the app completed predefined workflow steps in all simulated, operator-run sessions. The segmentation model produced outputs on all images, and the average session duration was 101.7 (SD 17.5) seconds. Simulated experience scores on a 5-point Likert scale were uniformly high.

**Conclusions:**

Glorbit is a cross-platform application that supports structured periorbital image capture and automated inference within a unified workflow. In simulated, operator-run testing, the platform demonstrated successful execution of predefined workflow steps across devices. These findings support the technical feasibility of the system as a research-oriented data collection framework and may inform future evaluations in broader research settings.

## Introduction

Periorbital measurements such as margin to reflex distance 1 and 2 (MRD 1/2), palpebral fissure height, and scleral show are essential components of clinical assessment in a range of conditions, including ptosis, thyroid eye disease, and congenital craniofacial conditions [1-3]. These measurements guide surgical decision-making, track disease progression, and serve as important inputs for diagnosis [4-7]. However, manual measurement of these distances incurs substantial intergrader variability, even when trained graders performed the analysis [8,9].

Previous work has demonstrated that automated periorbital measurements from facial photographs can achieve clinically acceptable accuracy by using deep learning–based segmentation approaches [10,11]. For example, a trained DeepLabV3 model achieved mean absolute errors consistently below established intergrader variability thresholds for key measurements, including MRD1, MRD2, and intercanthal distance, with 86% of the measurements falling within intergrader thresholds. This approach also outperformed the benchmark method PeriorbitAI, successfully processing 100% of the images across diverse disease populations, including thyroid eye disease and craniofacial conditions, compared to 59%-85% success rates for PeriorbitaAI [10,12]. However, due to the lack of deployable infrastructure capable of integrating image capture, processing, and secure data management, both of these algorithms (and others) cannot be integrated into routine data collection workflows for research use.

In response to this gap, we developed Glorbit, a web-based application for artificial intelligence (AI)-based periorbital distance measurement and metadata capture. Rather than introducing a new periorbital measurement algorithm, the primary contribution of Glorbit lies in system integration or in the unification of image capture, local preprocessing, AI inference, metadata collection, and secure, site-specific data storage within a browser-based interface. This contribution is orthogonal to algorithm development, and as such, we focus exclusively on assessing workflow operability of the application rather than measurement accuracy, reliability, or readiness for clinical use. In this paper, we describe the system architecture and image processing pipeline and present a small, simulated feasibility study assessing end-to-end workflow execution and participant-perceived enrollment experience under controlled conditions.

## Methods

### Ethical Considerations

The simulated enrollment study was designed to evaluate technical operability and workflow execution rather than performance under real world clinical constraints. No direct identifiers (eg, name, date of birth, medical record number) were collected, and each participant was assigned a deidentified patient ID. Verbal consent was obtained in accordance with an institutional review board–exempt protocol (STUDY2025-0731). All captured images, derived measurements, and metadata were deleted following data analysis. No participant images or metadata were retained in persistent cloud buckets. Participants were not compensated.

### System Overview and User Flow

**Figure 1:**
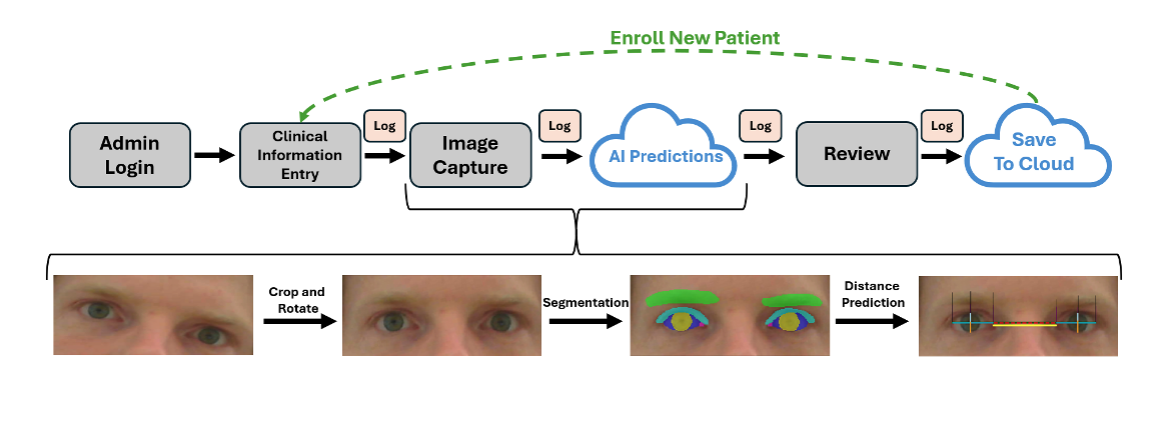
Glorbit application workflow and artificial intelligence (AI) processing pipeline. The top row illustrates the user-facing app interface: a user logs in, enters clinical metadata, captures an image, reviews AI-predicted periorbital distances, and uploads results to cloud storage. Cloud-shaped nodes denote steps involving remote model inference or cloud-based data storage. All user actions, including reverse navigation, are logged. The bottom row depicts the backend image processing steps: cropping and rotation based on facial landmarks, semantic segmentation of key anatomical regions, and automated distance prediction using landmark geometry.

Glorbit is a browser-based application designed to support structured periorbital image capture and automated distance inference for research use. Upon login, authenticated users are presented with a structured form to collect brief patient history and visit metadata. This information is stored temporarily in session state within the browser. After submission, users proceed to an image capture page, where a webcam or device camera is used to capture a frontal facial image. The image is processed locally to generate segmentation and AI-derived distance measurements, which are then displayed for user review. Once confirmed, the image and associated metadata are uploaded to a secure, site-specific cloud storage location. Logging is integrated throughout the pipeline to capture system events, errors, and upload status. See Figure 1 for an overview of users’ steps throughout the application.

### Image Processing Pipeline

Each captured image undergoes a predefined processing pipeline for anatomical alignment, cropping, and segmentation, as described by Nahass et al [10]. MediaPipe FaceMesh landmarks are used to rotate and align the image to a horizontal eye-level axis [13]. A region of interest is then cropped around the eyes and periorbital region. The aligned image is passed to a trained DeepLabV3 segmentation model for periorbital anatomic segmentation, followed by the geometric calculation of periorbital distances from the segmented output (Figure 1) [14]. In the event of a model failure, the app handles the error, logs the failure, and prompts the user to try again. The periorbital distance prediction model integrated into Glorbit was selected from prior work and was used without modification; evaluation of measurement accuracy, repeatability, and robustness was outside the scope of this study, which focused on platform feasibility and workflow execution.

### Clinical Data

Glorbit captures a structured set of clinical metadata alongside each image to support contextual analysis and downstream modeling. At minimum, users are required to input a deidentified patient ID, age, and sex to proceed with the workflow. Optional fields include ethnicity (selected from a site-customizable dropdown list), relevant comorbidities (eg, thyroid eye disease, craniofacial conditions, prior eyelid surgery), visit type (new or follow-up), and image conditions (eg, use of tape, lighting notes). Sites may also enable the entry of laboratory values when available. Supported laboratory measurements include levels of alanine aminotransferase, aspartate aminotransferase, hemoglobin A_1c_, estimated glomerular filtration rate, thyroid-stimulating hormone, thyroid-stimulating immunoglobulin, albumin-to-creatinine ratio, and calcium, with standardized units displayed next to each input. All metadata are stored alongside the captured image and the AI-derived periorbital distances in the designated site-specific cloud storage bucket. Only the required fields must be completed to advance to image capture; all other fields are optional and may be tailored to institutional needs.

### Data Handling and Storage Architecture

**Figure 2:**
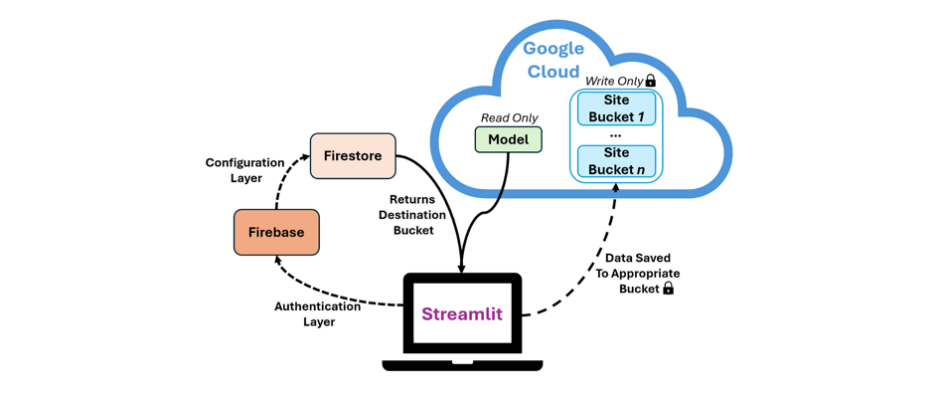
System architecture for secure authentication, configuration, and cloud-based storage within the Glorbit platform. Users authenticate via Firebase, after which Firestore provides site-specific configuration, including the destination storage bucket. The local Streamlit app reads artificial intelligence model weights from a centralized, read-only location in Google Cloud and writes collected data to site-specific, write-only buckets. All data transfers are protected using encryption at rest and in transit, as indicated by lock icons. Access control is governed via Google Cloud’s Identity and Access Management framework.

The app frontend is built in Streamlit and deployed as a Docker container to ensure reproducibility. Firebase Authentication manages secure login, with role-based access control and per-site credentialing. Firestore is used to assign each user’s upload path based on their authenticated credentials, enabling dynamic configuration of storage destinations. This allows a single instance of the app to be deployed across multiple locations without requiring hardcoded site-specific settings or local reconfiguration. Glorbit stores the following data elements: (1) cropped facial images, (2) AI-predicted periorbital measurements, (3) session-level metadata entered by the operator, and (4) system logs generated for debugging and audit. All data are encrypted at rest and in transit, and access rules are configured through Google Cloud Identity and Access Management policies. Glorbit is designed to operate on consumer-grade hardware and can be accessed on laptops, tablets, and mobile phones with integrated webcams. A graphical schematic of data movement from the operator’s perspective can be found in Figure 2.

### Security Design

The primary data elements with potential status as Protected Health Information within the Glorbit workflow are cropped images of the periorbital region. While the removal of the full face reduces identifiability, we treat these cropped images as sensitive biometric data. To protect this information, all uploads utilize technical safeguards, including encryption in transit (TLS) and at rest (AES-256) via Google Cloud’s native encryption [15-17]. Each deployment site is assigned a unique Google Cloud Storage bucket with Identity and Access Management roles that can be configured to restrict access by site. Firebase Authentication enforces user-specific logins, and Firestore assigns site-level write access dynamically. Full event and error logging is implemented for auditability. The platform architecture incorporates technical safeguards commonly required for Health Insurance Portability and Accountability Act (HIPAA)-compliant workflows; however, compliance is conditional upon the specific deployment environment. Specifically, when deployed within an institutional environment governed by a Business Associate Agreement with the cloud provider, the system provides the necessary infrastructure to maintain a HIPAA-compliant data pipeline when appropriately configured [18,19]. In the absence of a Business Associate Agreement between an institution deploying Glorbit and the cloud provider, Glorbit is not HIPAA-compliant.

### Internal Testing: Cross-Platform Compatibility

Glorbit was tested by a member of the study team (GRN) across multiple operating systems (Windows, iOS, ChromeOS), browsers (Chrome, Firefox, Safari), and device types (laptop, personal computer, smartphone, tablet) to assess basic cross-platform compatibility. Testing focused on verifying that core workflow components, including authentication, metadata entry, camera access, image capture, and execution of the image processing pipeline, functioned consistently across platforms. A workflow failure was defined as the inability to successfully complete any predefined step, including authentication errors, camera access failure, image capture failure, segmentation, or inference errors, resulting in missing outputs, upload errors, or logging failures. Failures were identified through user-facing error messages hardcoded into the pipeline, as well as through a review of system logs confirming completion of each stage. In addition, controlled negative-input scenarios such as the absence of a detectable face in the camera frame were manually tested to confirm appropriate system behavior, including user-facing feedback and event logging. These checks were performed to validate control flow and error handling rather than to quantify measurement accuracy. This testing was performed under natural indoor lighting conditions with 5 trial runs on each combination.

### External Testing: Survey and Simulated Enrollment Testing

To evaluate participant-perceived enrollment experience, we conducted a simulated enrollment study with 15 adult volunteers (age ≥18 years) at the Illinois Eye and Ear Infirmary. Each session was conducted by a trained study operator (GRN) using a 2021 MacBook Air in a private room under natural indoor lighting conditions. The operator followed the standard Glorbit workflow, including metadata entry and facial image capture. Failures of the workflow were assessed using the same protocol as described earlier.

Following the simulated interaction, participants completed a brief anonymous survey assessing their experience immediately following the interaction. The survey included 5 Likert-scale items (1-5) evaluating perceived intuitiveness, perceived efficiency, clarity of workflow steps, self-reported confidence in the displayed outputs, and perceived potential usefulness in a clinical context. All responses were stored without any identifiers. This survey was intended to capture participant-perceived workflow clarity and general impressions following an operator-run simulated interaction; it was not designed to assess operator usability, measurement validity, or clinical readiness. Any reported confidence in the displayed outputs following the simulated interaction reflect participant perception only and does not represent validation of measurement accuracy.

## Results

### App Walkthrough and Cross-Platform Compatibility

**Figure 3:**
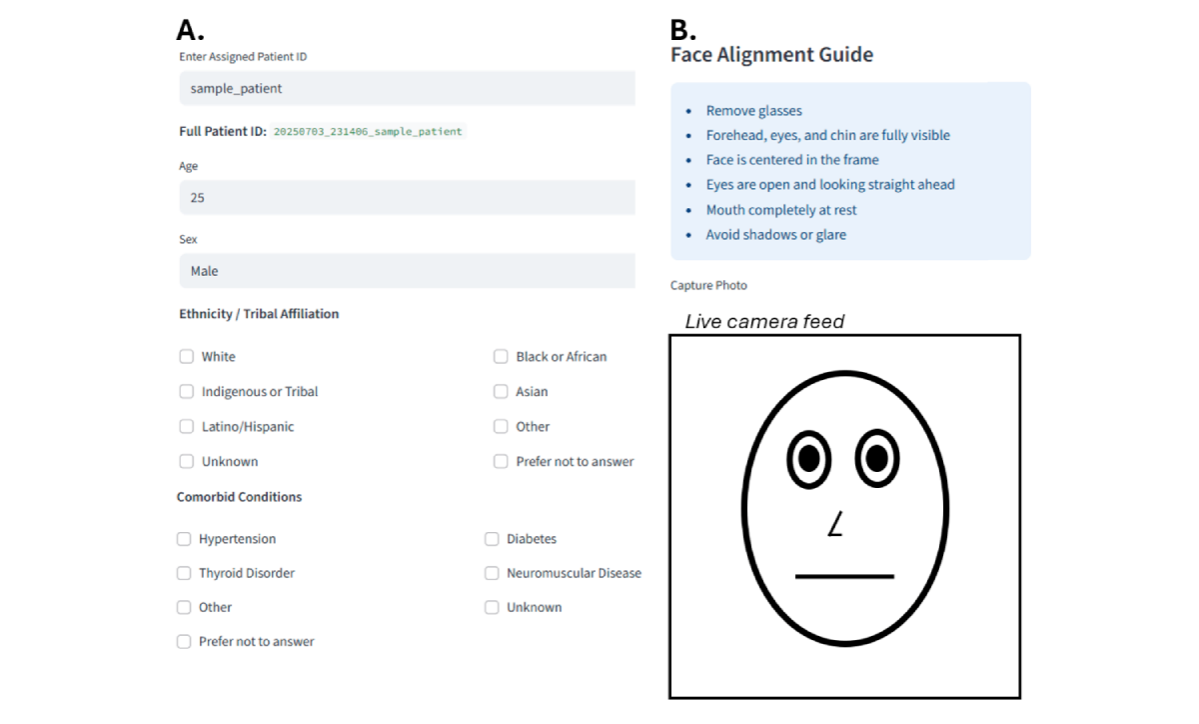
Screenshot of the Glorbit app interface. (A) The form used to input patient metadata, including demographics and comorbidities. (B) Face alignment guide with a live camera view for guided image capture. These two steps represent the only direct patient interactions aside from the final review and submission step.

Screenshots of the core workflow of Glorbit are shown in Figure 3, illustrating the minimalistic, stepwise interface. To assess platform execution, Glorbit was internally tested across multiple browsers, operating systems, and device types. Across all tested platforms, core workflow components, including authentication, form entry, camera access, image capture, execution of the image processing pipeline, and system logging, were executed without observed workflow interruption across tested configurations.

Table 1 shows the evaluated platforms and devices. In addition, controlled negative input scenarios were manually evaluated, including cases in which no face was detectable within the camera frame. In these scenarios, the system generated appropriate user-facing feedback and recorded the event in the application logs.

**Table 1:** Summary of the operating systems, device types, and camera sources that the workflow feasibility of Glorbit was evaluated on. Five execution runs were completed for each configuration.

	Operating system	Device type	Browser	Camera source
1	Windows	Desktop PC	Chrome	USB webcam
2	Windows	Desktop PC	Firefox	USB webcam
3	macOS	MacBook	Chrome	Integrated webcam
4	macOS	MacBook	Firefox	Integrated webcam
5	macOS	MacBook	Safari	Integrated webcam
6	ChromeOS	Chromebook	Chrome	Integrated webcam
7	iOS	Smartphone	Chrome	Front camera
8	iOS	iPad	Chrome	Front camera

### Simulated Enrollment Performance

We enrolled 15 adult volunteers in a simulated enrollment study to assess the workflow operability of Glorbit under simulated operating conditions. Participant demographics are summarized in Table 2. The group’s mean age was 32.6 (SD 11.0) years, was 67% (10/15) female, and spanned 5 self-identified ethnic groups.

In all 15 cases (100%), the app completed the predefined workflow steps (metadata entry, image capture, segmentation, and upload) in all the simulated sessions conducted by a trained operator. The segmentation model generated outputs in all cases, and no image processing or upload failures were observed under the tested conditions. The average session duration, measured from initial form entry to final upload, was 101.7 (SD 17.5) seconds on consumer-grade hardware (Figure S1 in Multimedia Appendix 1). Logging was successful in all cases, with system events and session metadata stored as expected.

**Table 2:** Demographic characteristics of the simulated study participants (N=15).

Characteristics		Values
**Age (y)**		
	Mean (SD)	32.6 (11.0)
	Maximum	58
	Minimum	24
	Median	26
**Sex, n (%)**		
	Male	5 (33)
	Female	10 (67)
**Ethnicity, n (%)**		
	White	2 (13)
	Latino/Hispanic	4 (27)
	Black or African	3 (20)
	Asian	5 (33)
	Other	1 (7)

### Participant Perception of Glorbit

Following each session, participants completed a brief anonymous postusage survey to assess their perceived experience during the simulated interaction. These responses reflect participant perceptions following an operator-run simulated interaction and do not represent an evaluation of clinician usability, workflow performance in routine practice, or validation of measurement accuracy. Average responses were high across all 5 items (Table 3).

Participants rated the app as intuitive and efficient (both 5.0, SD 0.0), with high self-reported confidence in the displayed outputs (4.9, SD 0.3) and a high perceived comfort in a hypothetical clinical context (4.9, SD 0.3). Participants also reported clear understanding of each step in the data collection workflow (4.8, SD 0.4). Across all participant responses to all questions, 93.3% (70/75) of the responses were 5, 6.7% (5/75) of the responses were 4, and no 3 or lower were observed. The implications of this ceiling effect are discussed in the Limitations section.

**Table 3:** Summary of participant feedback from postsession surveys in the simulated enrollment study. Each participant (N=15) rated 5 statements on a 5-point Likert scale (1=strongly disagree, 5=strongly agree).

Statement	Average rating, mean (SD)
The Glorbit app was intuitive and easy to use.	5.0 (0.0)
The process of capturing and uploading patient data was fast and efficient.	5.0 (0.0)
I would feel comfortable using this tool in a clinical setting.	4.9 (0.3)
I understood each step of the data collection process.	4.8 (0.4)
I have confidence in the displayed outputs.	4.9 (0.3)

## Discussion

### Principal Findings

Many periorbital distance prediction algorithms have been developed in recent years, but their utility as broader research tools remains limited by the absence of deployable infrastructure [9,12,20-22]. In such contexts, there is a need for lightweight and secure systems capable of image capture, processing, and storage that can be employed within structured data collection workflows. To meet this need, we developed an internet browser–based app, Glorbit. Glorbit does not introduce a new periorbital measurement algorithm; instead, its primary contribution lies in the integration of existing periorbital measurement models into a unified system that supports image capture, inference, metadata collection, and secure storage. Periorbital distance measurement is a property of the underlying AI model rather than the deployment platform itself, and therefore, evaluation of this was outside the scope of this study, which was designed to assess the workflow operability of Glorbit.

In a simulated study of 15 participants, Glorbit completed predefined workflow steps (metadata entry, image capture, segmentation, and upload) in all simulated sessions conducted under operator-run conditions. These findings reflect successful technical execution of the workflow under simulated conditions and should not be interpreted as evidence of field readiness or validated clinical usability. The uniformly high survey ratings reflect participant-perceived workflow clarity following an operator-run simulated interaction and do not represent an evaluation of clinician usability, workflow performance in routine practice, or clinical utility. Glorbit provides error messages and logs all user movement in the event of a failure (which were not observed in the simulated user study). This error handling is intended to provide administrators with detailed diagnostic information for troubleshooting workflow interruptions during future testing or deployment.

Additionally, the complete end-to-end workflow duration through Glorbit was short, with a mean session time of 101.7 (SD 17.5) seconds, inclusive of clinical metadata entry and user review of AI generated outputs. For contextual reference, prior studies have reported longer durations for traditional periorbital measurement workflows, including assisted measurement using ImageJ toolkits (327, SD 116 s) and purely manual measurement (804, SD 204 s) [23]. These previously reported values were obtained under different study designs and are provided here for qualitative context rather than as a direct efficiency comparison. Additionally, these timing results were obtained during simulated, operator-run enrollments and may not generalize to routine clinical or real-world research settings.

To date, several mobile apps have been developed for facial measurement. However, these tools primarily focus on interpupillary distance and do not include integrated metadata capture or cloud upload functionality for research workflows [24,25]. Glorbit is designed to include a combination of site-specific secure cloud storage and a modular architecture that can be deployed across institutions without requiring local IT infrastructure. Table 4 summarizes selected functional characteristics of Glorbit and other facial measurement tools, emphasizing deployment features rather than measurement accuracy or clinical validation.

**Table 4:** Comparison of Glorbit to existing facial measurement platforms.

Platform	Cloud storage^a^	Measurement type^b^	Open source	Primary function
Glorbit	Yes	MRD^c^1, MRD2, PFH^d^ (48 distances)	Yes	Integrates inference, metadata forms, and storage
MediaPipe [13]	No	Facial landmarks	Yes	Google tool for face mesh detection
OpenFace [26,27]	No	Facial landmarks	Yes	Academic tool for facial behavior analysis, can extract 68 landmarks

^a^Cloud storage reflects availability of integrated cloud upload functionality.

^b^Measurement type describes the anatomical features or distances quantified by each system.

^c^MRD: margin to reflex distance.

^d^PFH: palpebral fissure height.

In addition to serving as a storage repository for providers, the captured data could potentially be used as future training data to create patient-specific models. One of the most persistent barriers to equitable AI is poor model generalization to underrepresented populations, often due to data captured under different conditions being out of distribution to the original training set [28-32]. Glorbit is designed to facilitate ethically governed data collection across diverse sites, which may support future efforts to improve model generalization. If integrated into research workflows, structured image and metadata capture could support the development of more representative, generalizable models through real-world data acquisition and iteration. Further strengthening the motivation for large-scale collection of high quality data is the developing field of Oculomics, which aims to leverage deep learning to make predictions about systemic health from images of the eyes [33-37]. While most current Oculomics research focuses on retinal imaging, recent studies have shown that external eye photographs can also be used to predict systemic biomarkers such as alanine aminotransferase, aspartate aminotransferase, and hemoglobin A_1c_ [38-40]. With the increasing number of foundational ophthalmic deep learning systems, having specific multimodal datasets available for finetuning may support future development and evaluation of downstream predictive models.

An important consideration for potential future deployment of tools like Glorbit in clinical settings is the protection of patient privacy and compliance with institutional and national data governance requirements. While the platform utilizes cropped periorbital images to minimize identifiability, these data elements are treated as sensitive biometric information requiring protection. Consequently, deployment within a regulated clinical environment would require the establishment of a Business Associate Agreement between the deploying institution and the cloud service provider to ensure HIPAA security requirements are met. Glorbit’s architecture is designed to incorporate technical safeguards (access control, end-to-end encryption, and audit logging) commonly required for HIPAA-compliant workflows when governed by such an agreement [18,19]. However, institutional oversight remains a necessary component of medical AI deployment, as the software's compliance status is conditional upon the host site’s administrative and security configurations. To promote transparency and allow for local adaptation and compliance with patient protection laws on a per nation basis, Glorbit is fully open-source.

### Limitations

This study represents an initial feasibility evaluation and should be interpreted in that context. The simulated enrollment involved a modest number of participants (N=15) and was designed to assess workflow operability, system stability, and platform execution rather than to formally evaluate usability using standardized instruments or to assess performance across diverse clinical users and settings. As such, measurement validity, repeatability, and robustness of the underlying periorbital distance prediction models were not assessed here. Although the simulated enrollment cohort included participants from multiple self-identified ethnic groups, the small sample size precluded meaningful subgroup analysis as well as drawing conclusions regarding reliability in real-world settings. Taken together, this study does not assess demographic-specific measurement accuracy, robustness across lighting conditions, or device-related biases—all important factors in computer vision applications. These factors represent known risks for facial analysis systems and will require dedicated, adequately powered evaluations in future studies.

In addition, sessions were conducted by a trained operator on a convenience sample, and participant feedback reflects perceived clarity and comfort following observed system interaction. As such, these ratings should be interpreted as indicators of basic workflow acceptability rather than sensitive or discriminative measures of usability performance and may not generalize to a larger population. Additionally, survey responses demonstrated strong ceiling effects, likely reflecting the controlled, operator-run nature of the simulated enrollment sessions. Future assessments of user experience (as opposed to participants) should incorporate standardized usability instruments and comparative study designs.

Finally, operator-facing usability and workflow burden were not formally evaluated in this study and would require evaluation in clinician-operated studies. Similarly, while the system architecture was engineered to permit offline use with local data storage, these capabilities were not evaluated here. Future work should include larger, clinician-operated evaluations across multiple sites to further assess usability and performance under routine clinical conditions.

## Appendix

Multimedia Appendix 1 Distribution of end-to-end session duration. Histogram showing the distribution of total session duration for simulated Glorbit enrollment sessions (N=15), measured from initial metadata entry through image capture, artificial intelligence (AI) processing, and final review.

## Abbreviations

AI: artificial intelligence
HIPAA: Health Insurance Portability and Accountability Act
MRD 1/2: margin reflex distance 1 and 2

## References

[1] Conrady C, Patel B (2025). Crouzon Syndrome.

[2] Koka K, Patel B (2023). Ptosis Correction.

[3] MacLachlan C, Howland HC (2002). Normal values and standard deviations for pupil diameter and interpupillary distance in subjects aged 1 month to 19 years. Ophthalmic Physiol Opt.

[4] Nemet AY (2015). Accuracy of marginal reflex distance measurements in eyelid surgery. J Craniofac Surg.

[5] Cruz AA, Coelho RP, Baccega A, Lucchezi MC, Souza AD, Ruiz EE (1998). Digital image processing measurement of the upper eyelid contour in Graves disease and congenital blepharoptosis. Ophthalmology.

[6] Cruz AA, Lucchezi MC (1999). Quantification of palpebral fissure shape in severe congenital blepharoptosis. Ophthalmic Plast Reconstr Surg.

[7] Bodnar ZM, Neimkin M, Holds JB (2016). Automated ptosis measurements from facial photographs. JAMA Ophthalmol.

[8] Boboridis K, Assi A, Indar A, Bunce C, Tyers AG (2001). Repeatability and reproducibility of upper eyelid measurements. Br J Ophthalmol.

[9] Hussey VM, Tao JP (2022). Oculofacial plastic surgeon distribution by county in the United States, 2021. Orbit.

[10] Nahass GR, Koehler E, Tomaras N, Lopez D, Cheung M, Palacios A, Peterson JC, Hubschman S, Green K, Purnell CA, Setabutr P, Tran AQ, Yi D (2025). State-of-the-art periorbital distance prediction and disease classification using periorbital features. ArXiv. Preprint posted online on May 14, 2025.

[11] Nahass GR, Koehler E, Tomaras N, Lopez D, Cheung M, Palacios A, Peterson JC, Hubschman S, Green K, Purnell CA, Setabutr P, Tran AQ, Yi D (2025). Open-source periorbital segmentation dataset for ophthalmic applications. Ophthalmol Sci.

[12] Van Brummen A, Owen JP, Spaide T, Froines C, Lu R, Lacy M, Blazes M, Li E, Lee CS, Lee AY, Zhang M (2021). PeriorbitAI: artificial intelligence automation of eyelid and periorbital measurements. Am J Ophthalmol.

[13] Kartynnik Y, Ablavatski A, Grischenko I, Grundmann M (2019). Real-time facial surface geometry from monocular video on mobile GPUs. ArXiv. Preprint posted online on July 15, 2019.

[14] Chen LC, Papandreou G, Schroff F, Adam H (2017). Rethinking atrous convolution for semantic image segmentation. ArXiv. Preprint posted online on December 5, 2017.

[15] Google security overview. Google Cloud.

[16] Default encryption at rest. Google Cloud.

[17] Encryption in transit for Google cloud. Google Cloud.

[18] Business associates. US Department of of Health and Human Services.

[19] HIPAA compliance on Google cloud. Google Cloud.

[20] Rana K, Beecher M, Caltabiano C, Macri C, Zhao Y, Verjans J, Selva D (2025). Artificial intelligence to automate assessment of ocular and periocular measurements. Eur J Ophthalmol.

[21] Chen H, Tzeng S, Hsiao Y, Chen R, Hung E, Lee OK (2021). Smartphone-based artificial intelligence-assisted prediction for eyelid measurements: algorithm development and observational validation study. JMIR Mhealth Uhealth.

[22] Guo Y, Liu J, Ruan Y, Rokohl A, Hou X, Li S, Jia R, Koch K, Heindl L (2021). A novel approach quantifying the periorbital morphology: a comparison of direct, 2-dimensional, and 3-dimensional technologies. J Plast Reconstr Aesthet Surg.

[23] Peterson JC, Nahass GR, Lasalle C, Bradley DC, Wu D, Zorra I, Nguyen A, Choudhary A, Heinze K, Purnell CA, Setabutr P, Yi D, Tran AQ (2025). Development and validation of a semiautomated tool for measuring periorbital distances. Ophthalmol Sci.

[24] Han KD, Jaafar M, Stoakes IM, Hoopes PC, Moshirfar M (2023). Comparing the effectiveness of smartphone applications in the measurement of interpupillary distance. Cureus.

[25] Singman E, Matta N, Tian J, Silbert D (2014). The accuracy of the plusoptiX for measuring pupillary distance. Strabismus.

[26] Baltrusaitis T, Robinson P, Morency L (2016). OpenFace: an open source facial behavior analysis toolkit.

[27] Hu J, Mathur L, Liang P, Morency L-P (2025). OpenFace 3.0: a lightweight multitask system for comprehensive facial behavior analysis. ArXiv. Preprint posted online on June 3, 2025.

[28] Hong Z, Yue Y, Chen Y, Cole L, Lin H, Luo Y, Wang MH, Wang W, Xu J, Yang X, Chen H, Li Z, Xie S (2024). Out-of-distribution detection in medical image analysis: a survey. ArXiv. Preprint posted online on July 3, 2024.

[29] Rashidisabet H, Sethi A, Jindarak P, Edmonds J, Chan RVP, Leiderman YI, Vajaranant TS, Yi D (2023). Validating the generalizability of ophthalmic artificial intelligence models on real-world clinical data. Transl Vis Sci Technol.

[30] Mårtensson Gustav, Ferreira D, Granberg T, Cavallin L, Oppedal K, Padovani A, Rektorova I, Bonanni L, Pardini M, Kramberger MG, Taylor J, Hort J, Snædal Jón, Kulisevsky J, Blanc F, Antonini A, Mecocci P, Vellas B, Tsolaki M, Kłoszewska I, Soininen H, Lovestone S, Simmons A, Aarsland D, Westman E (2020). The reliability of a deep learning model in clinical out-of-distribution MRI data: a multicohort study. Med Image Anal.

[31] Ozkan E, Boix X (2024). Multi-domain improves classification in out-of-distribution and data-limited scenarios for medical image analysis. Sci Rep.

[32] Karimi D, Gholipour A (2023). Improving calibration and out-of-distribution detection in deep models for medical image segmentation. IEEE Trans Artif Intell.

[33] Zhu Z, Wang Y, Qi Z, Hu W, Zhang X, Wagner SK, Wang Y, Ran AR, Ong J, Waisberg E, Masalkhi M, Suh A, Tham YC, Cheung CY, Yang X, Yu H, Ge Z, Wang W, Sheng B, Liu Y, Lee AG, Denniston AK, Wijngaarden PV, Keane PA, Cheng C, He M, Wong TY (2025). Oculomics: current concepts and evidence. Prog Retin Eye Res.

[34] Honavar SG (2022). Oculomics - the eyes talk a great deal. Indian J Ophthalmol.

[35] Patterson EJ, Bounds AD, Wagner SK, Kadri-Langford R, Taylor R, Daly D (2024). Oculomics: a crusade against the four horsemen of chronic disease. Ophthalmol Ther.

[36] Suh A, Hampel G, Vinjamuri A, Ong J, Kamran SA, Waisberg E, Paladugu P, Zaman N, Sarker P, Tavakkoli A, Lee AG (2024). Oculomics analysis in multiple sclerosis: current ophthalmic clinical and imaging biomarkers. Eye (Lond).

[37] Zhou Y, Chia MA, Wagner SK, Ayhan MS, Williamson DJ, Struyven RR, Liu T, Xu M, Lozano MG, Woodward-Court P, Kihara Y, Altmann Andre, Lee Aaron Y, Topol Eric J, Denniston Alastair K, Alexander Daniel C, Keane Pearse A (2023). A foundation model for generalizable disease detection from retinal images. Nature.

[38] Babenko B, Traynis I, Chen C, Singh P, Uddin A, Cuadros J, Daskivich LP, Maa AY, Kim R, Kang EY, Matias Y, Corrado GS, Peng L, Webster DR, Semturs C, Krause J, Varadarajan AV, Hammel N, Liu Y (2023). A deep learning model for novel systemic biomarkers in photographs of the external eye: a retrospective study. Lancet Digit Health.

[39] DeBuc DC (2023). AI for identification of systemic biomarkers from external eye photos: a promising field in the oculomics revolution. Lancet Digit Health.

[40] Babenko B, Mitani A, Traynis I, Kitade N, Singh P, Maa AY, Cuadros J, Corrado GS, Peng L, Webster DR, Varadarajan A, Hammel N, Liu Y (2022). Detection of signs of disease in external photographs of the eyes via deep learning. Nat Biomed Eng.

