# Supplementary figures and images for: Simulated Workflow Feasibility Evaluation of a Web-Based Periorbital Measurement Platform: Development and Usability Study
Source: JMIR Hum Factors. 2026 Apr 17;13:e82859. doi: 10.2196/82859 (PMC13135169; doi:10.2196/82859)

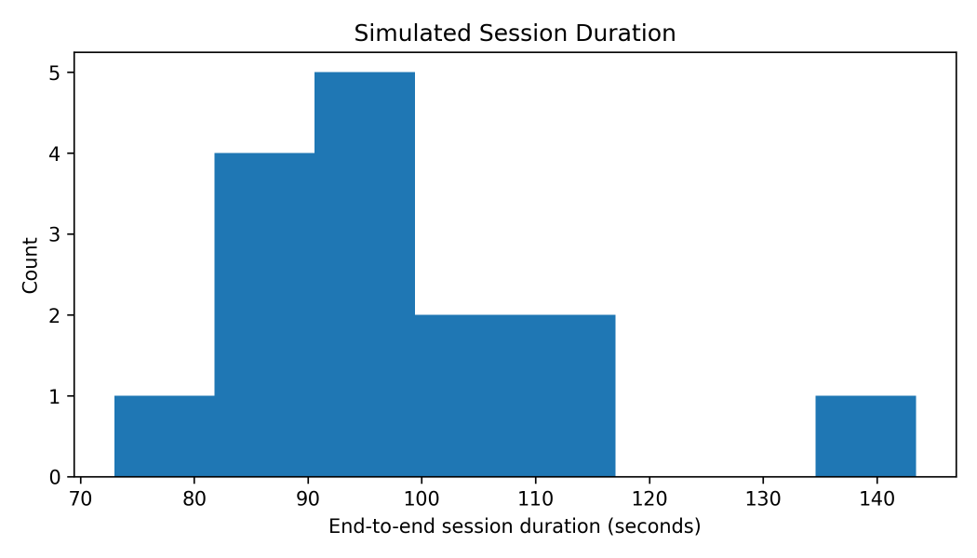

Supplement: Multimedia Appendix 1 [file humanfactors_v13i1e82859_app1.png]
